# Supplementary material for: The Associations Between Older Driver Licensure Laws with Travel and Passenger Behaviors Among Adults Aged 65 Years or Older (United States, 2003–2017)
Source: Int J Environ Res Public Health. 2021 Feb 25;18(5):2251. doi: 10.3390/ijerph18052251 (PMC7956313; doi:10.3390/ijerph18052251)
Supplement: Supplementary file 1 [file ijerph-18-02251-s001.pdf]

Table S1. Sensitivity analysis on the coding of in-person renewal period for adults not required to renew their license in-person.

Gender	Coding Method	65–74 Years		75 Years or Older	
		Travel	Passenger	Travel	Passenger
Male	Removed	1.00 (0.99–1.00)	1.00 (0.95–1.04)	1.00 (0.99–1.02)	0.98 (0.94–1.01)
	Coded as 30	1.00 (1.00–1.00)	1.02 (1.00–1.04)	1.00 (1.00–1.01)	0.99 (0.97–1.01)
	Coded as 35	1.00 (1.00–1.00)	1.02 (1.00–1.04) *	1.00 (1.00–1.01)	0.99 (0.97–1.01)
Female	Removed	1.00 (0.99–1.00)	0.99 (0.97–1.01)	1.00 (0.99–1.01)	1.01 (0.99–1.04)
	Coded as 30	1.00 (0.99–1.00)	0.99 (0.98–1.01)	1.00 (0.99–1.00) **	1.00 (0.99–1.01)
	Coded as 35	1.00 (1.00–1.00)	1.00 (0.99–1.00)	1.00 (0.99–1.00) **	1.00 (0.99–1.01)

Note: * indicates statistical significance at 0.05; ** indicates statistical significance at 0.025 (0.05/2), 97.5% CI.

Table S2. Provisions of state driver license renewal laws over the study period, 2003–2017.

State	Renewal Cycle [a]	Accelerated Renewal Cycle for Older Drivers [a]	In-person Renewal Frequency [b]	Accelerated In-Person Renewal Frequency for Older Adults [b]	Other Renewal Provisions
Alabama	4	No	2	No	None
Alaska	5	No	2	1 for age 69 and older	Vision test required for all ages
Arizona	12	5 for age 65 and older	None	1 for age 70 and older	Vision test required for all ages
Arkansas	8, 4 before Jan 2016	No	1	No	Vision test required for all ages
California	5	No	3	1 for age 70 and older	Vision test required for all ages; Knowledge test for age 70 and older; Mandatory reporting laws for physicians for all ages
Colorado	5, 10 before May 2005	No, 5 for 61 and older before May 2005	3, 2 before Aug 2008	2 for age 66 and older, 1 for age 61 and older between May 2006 and Aug 2008, and	Vision test required for all ages; Vision test report required if no in-person vision test is conducted
Connecticut	6, 4 before July,2011	2 for age 65 and older	2, 1 before July 2011	No	Vision test required for all ages before July 2007.
Delaware	8, 5 before Dec 2011	No	1	No	Vision test required for all ages; Mandatory reporting laws for physicians for all ages
Dist. Of Columbia	8, 5 before Sep 2008	No	2	1 for age 70 and older	Vision test required for age 70 and older; Mandatory reporting laws for physicians for age 70 and older
Florida	8, 6 before Oct 2008	6, No before Oct 2008	2, 3 before Oct 2008	No	Vision test required for all ages;
Georgia	8, 5 before May 2016, 4 before July 2005	5 for age 65 and older	2	1 for age 65 and older	Vision test required for age 65 and older after July 2005; Vision test report required if no in-person vision test is conducted
Hawaii	8, 6 before Nov 2008	2 for age 72 and older	3	No	Vision test required for all ages
Idaho	4 or 8	4 for age 63 and older	2	1 for age 70 and older	Vision test required for all ages
Illinois	4	2 for ages 81–86 and 1 for ages 87 and older	2	1 for age 75 and older	Vision test required for all ages; Knowledge test required for all ages; On-road test for age 75 and older
Indiana	6, 4 before	3 for ages 75–84, 2 for	2	1 for age 75 and older, 1 for age 70	Vision test required for all ages;

	Jan 2006	ages 85 and older, and 3 for ages 85 and older before July 2005		and older between July 2010 and July 2012, and No before July 2010	Knowledge test required for age 75 and older before July 2004; On-road test for age 75 and older before July 2005
Iowa	5–8, 5 before Jan 2014	2 for ages 72 and older; 2 for ages 70 and older before May 2013	1	No	Vision test required for all ages
Kansas	6	4 for ages 65 and older	1	No	Vision test required for all ages; Knowledge test required for all ages before July 2010
Kentucky	4	No	1	No	
Louisiana	6, 4 before July 2015	No	2	1 for age 70 and older	Vision test required for all ages
Maine	6	4 for ages 65 and older	2	1 for age 62 and older	Vision test required for all ages
Maryland	5, 8 before Oct 2015, 5 before Oct 2012	No	2	No	Vision test required for all ages
Massachu-setts	5	No	2	No	Vision test required for all ages
Michigan	4	No	2	No	Vision test required for all ages; Knowledge test required for all ages before June 2003
Minnesota	4	No	1	No	Vision test required for all ages
Mississippi	4	No	2, not required before July 2005	1 for age 75 and older	
Missouri	6	3 for ages 70 and older	1	No	Vision test required for all ages
Montana	8	4 for ages 75 and older	2, not required between Oct 2005 and Jan 2017 2 before Oct 2005	1 for age 75 and older, No before Oct 2005	Vision test required for all ages
Nebraska	5	No	2, 1 before April 2010	1 for ages 72 and older, No before Aug 2011	Vision test required for all ages
Nevada	8, 4 before Jan 2014	4 for ages 65 and older	2, 1 before Aug 2008	No	Vision test required for all ages; Vision test report required if no in-person vision test is con-ducted; Mandatory reporting laws for physicians for age 70 and older
New Hampshire	5	No	2, 1 before Aug 2008	No	Vision test required for all ages; On-road test for age 75 and older before July 2011
New Jersey	4	2 for ages 70 and older	2, 1 before Jan 2012	No	Mandatory reporting laws for physicians for all ages
New Mexi-co	4 or 8	1 for ages 79 and older; 1 for ages 75 and older before May 2016; 4 for ages 67-74 before May 2016	2, 1 before July 2004	1 for age 75 and older, No before July 2004	Vision test required for all ages
New York	8	No	1	No	Vision test required for all ages
North Car-olina	8, 5 before Jan 2007	5 for ages 66 and older; 5 for ages 54-65 between Jan 2007 and Jan 2011	1	No	Vision test required for all ages
North Da-	6, 4 before	4 for ages 78 and older	1	No	Vision test required for all ages

kota	July 2011				
Ohio	4	No	1	No	Vision test required for all ages
Oklahoma	4	No	1	No	
Oregon	8	No	1, 2 before Oct 2004	No	Vision test required for age 50 and older after June 2003; Mandatory reporting laws for physicians for all ages
Pennsylvania	4	No	Not required	No	Mandatory reporting laws for physicians for all ages
Rhode Island	5	2 for ages 75 and older; 2 for ages 70 and older before July 2008	2, 1 before Jan 2012	1 for age 75 and older, No before Jan 2012	Vision test required for all ages
South Carolina	10, 5 before Oct 2003	5 for ages 65 and older	1, 2 before Oct 2003	2 for age 65 and older	Vision test required for age 65 and older; Vision test required for all ages after Oct 2008
South Dakota	5	No	1	No	Vision test required for all ages
Tennessee	5	No	2	No	
Texas	6	2 for ages 85 and older after Jan 2007	2	1, No before Sep 2007	Vision test required for all ages
Utah	5	No	1, 2 before Dec 2009	No	Vision test required for age 65 and older after Jan 2011
Vermont	2 or 4, 4 before Jan 2014	No	2, 1 before June 2004	No	
Virginia	8, 5 before Jan 2009	5 for ages 75 and older after Jan 2015	2	1 for ages 75 and older, 1 for ages 80 and older before Jan 2015, No before Jan 2005	Vision test required for all ages
Washington	6, 5 before Jan 2012	No	2, 1 before Nov 2004	1 for ages 70 and older, No before Nov 2004	Vision test required for all ages
West Virginia	8, 5 before June 2014	No	1	No	Vision test required for all ages after March 2008
Wisconsin	8	No	1	No	Vision test required for all ages
Wyoming	4	No	2	No	Vision test required for all ages

Note. [a] The unit of renewal cycle is in years; [b] In-person renewal frequency: 1 = every renewal period, 2 = every other renewal period, 3 = every third renewal period.

As of 2017, out of the 50 states and District of Columbia (DC), 22 states required drivers over a specific age to renew their license with a shorter renewal period than younger adults. In 18 states and DC, older drivers must renew in-person while younger drivers might renew online or by mail. All drivers must pass a vision test at in-person renewals in 39 states and DC, but 3 states (Colorado, Florida, and Nevada) require drivers to pass a vision test if an in-person renewal was not conducted (e.g., drivers are required to submit a vision report from their healthcare providers). Older drivers in three states (Illinois, Indiana, and New Hampshire) must pass an on-road test to renew their licenses (Indiana and New Hampshire repealed on-road testing in 2005 and 2011, respectively), and five states (California, Illinois, Indiana, Kansas, and Michigan) required or had required all or older drivers to take a knowledge test for license renewal. From 2003 to 2017, drivers in six states (California, Delaware, New Jersey, Nevada, Oregon, and Pennsylvania) and DC were subject to mandatory reporting laws which required physicians to report their patients to the licensing authority if certain conditions occurred (reported conditions varied by state).
